# Investigating the role of introns in the regulation of regenerating gene 1 expression

**DOI:** 10.3892/ol.2014.2712

**Published:** 2014-11-19

**Authors:** YURONG CHAI, YUN SUN, LINXIA GUO, DAN LI, YI DING

**Affiliations:** Department of Histology and Embryology, College of Basic Medicine, Zhengzhou University, Zhengzhou, Henan 450001, P.R. China

**Keywords:** human gastric cancer, regenerating gene 1, gastrin, intron, transcription factors

## Abstract

Gastrin is a hormone that physiologically regulates gastric acid secretion and contributes to the maintenance of gastric epithelial architecture by regulating the expression of genes such as regenerating gene 1 (*Reg1*). Reg1 is involved in gastric carcinogenesis as an antiapoptotic factor. The current study explores the molecular mechanism of gastrin-regulated *Reg1* expression in human gastric cancer cells. In total, five intron fragments of the *Reg1* gene were cloned by polymerase chain reaction and inserted into luciferase reporter vector pGL3 to construct intron-luciferase reporter vectors. After confirmation by Xho I/Hind III digestion and DNA sequencing, the five constructs were transfected into the SGC7901 gastric cancer cell line. The luciferase activity of the cells transfected with each of the five constructs was detected following incubation without or with gastrin. The five intron fragments of *Reg1* were also randomly labeled with digoxin as a probe, and nuclear proteins of gastric cancer cells were extracted following treatment with or without gastrin. Southwestern blotting was subsequently performed to detect transcription factors that bind to the introns. The results indicated that the luciferase activity was significantly higher in cells transfected with recombinant vectors containing introns 2, 3, 4 or 5 than that in the cells transfected with an empty vector (P<0.05). However, no statistically significant difference in luciferase activity was identified between cells transfected with pGL3-intron 1 and those transfected with pGL3-Basic (P>0.05). Following incubation with gastrin, no significant difference was identified (P>0.05). The five introns of *Reg1* can bind a number of transcription factors and gastrin may affect this interaction. Introns 2–5 of *Reg1* potentially have transcriptional control over gene expression in gastric cancer cells. In conclusion, gastrin may regulate the expression of the *Reg1* gene via the interaction of the introns by binding to the transcription factors.

## Introduction

Gastric cancer (GC) is a major cause of cancer-related mortalities worldwide (1). There are multiple pathogenic factors that may promote the development and progression of gastric cancer. Gastrin is a type of polypeptide hormone, secreted by G cells in the stomach and upper-section of the small intestines. Its role in the physiological regulation of gastric acid secretion has been well established (2). Additionally, gastrin contributes to the maintenance of gastric epithelial architecture by regulating the expression of genes such as plasminogen activator inhibitor 1 (PAI-1) (3) and regenerating gene 1 (*Reg1*) (4). Increased expression of gastrin has been demonstrated in the progression of intestinal gastric cancer (5).

*Reg1* encodes a β-cell growth factor, Reg1 protein, primarily observed during pancreatitis and pancreatic islet regeneration (6,7). Reg1 has also been referred to as pancreatic stone protein (PSP) (8), or lithostathine (9), according to different studies. The Reg family comprises of four subclasses (Reg1–4) (10) across species, with the majority of the orthologs belonging to the Reg1 and Reg3 groups. The *Reg1* gene is approximately 3 kb in size, containing six exons and five introns, and is located at chromosome 2p12. An increasing number of studies have provided evidence of the participation of Reg1 protein in the proliferation and differentiation of diverse cell types (11,12). It has been demonstrated that Reg family members are associated with various pathologies, including diabetes, epithelial inflammation and a number of forms of cancer (13,14). *Reg1* is predominantly expressed in enterochromaffin-like (ECL) cells, as well as pepsinogen-secreting chief cells, which are also a target of gastrin within the gastric epithelium (15). It has been proposed that Reg1 and gastrin may synergistically regulate gastric mucosal proliferation during certain pathological settings (16,17). Significantly less is known with regard to the transcriptional mechanisms by which gastrin may regulate genes involved in the maintenance of gastric epithelial architecture. It was previously reported that a C-rich region of the proximal promoter sequence of *Reg1* is required for gastrin-stimulated expression in gastric cancer cell line, AGS (15), and that the expression of *Reg1* is controlled through separate promoter elements −2111 and −104 bp by gastrin (4).

It has been demonstrated that intronic sequences in eukaryotes have the potential to improve gene expression through a variety of mechanisms. Human β-globin (hBG) introns can act as enhancer-like elements for the expression of the human factor *IX* in cultured Chinese hamster ovary cells, resulting in a higher activity with respect to the second hBG intron compared with the first one. The larger number of transcription factor binding motifs in the second hBG intron accounts for its stronger effect (18). The DNase I-hypersensitive (HSS) sequences in intron 51 of the von Willebrand factor (VWF) gene contain *cis*-acting elements that are necessary for the VWF gene transcription in a subset of lung endothelial cells *in vivo* (19). It was demonstrated that Reg1 and gastrin may synergistically regulate gastric mucosal proliferation during certain pathological settings such as wound healing (16). To identify additional *cis*-acting elements within the *Reg1* gene that may participate in transcriptional regulation and the effects of gastrin on expression of *Reg1*, we investigated whether introns of *Reg1* gene can increase its expression and bind to transcription factors in gastric cancer cells. This also facilitated the exploration of the cellular mechanisms regulating *Reg1* expression in gastric cancer cells.

## Materials and methods

### Cell culture

The human gastric cancer cell line, SGC7901, was provided by the Cell Bank of the Chinese Academy of Sciences (Shanghai, China) and routinely cultured in RPMI 1640 medium (Gibco, Grand Island, NY, USA) supplemented with 10% fetal bovine serum, 100 U/ml penicillin (Sigma-Aldrich, St. Louis, MO, USA) and 100 μg/ml streptomycin (Sigma-Aldrich), at 37°C in a humidified atmosphere of 5% CO_2_.

### Cloning of the human Reg1 gene introns

In total, five intron fragments of *Reg1* were cloned from genomic DNA of human blood cells by polymerase chain reaction (PCR). PCR was performed in a final volume of 25 μl. The PCR reaction was carried out at 94°C for 3 min, followed by 35 cycles at 95°C for 30 sec, 50–55°C for 45 sec and 72°C for 1 min. PCR primers for the five introns are listed in Table I. The PCR fragments of five introns were inserted into pbluescript II SK+, digested by Xho I/Hind III (Takara Biotechnology Co., Ltd., Dalian, China) and subcloned into luciferase reporter vector pGL3-Basic, which was used as a control and digested with the same two enzymes. The five luciferase reporter vectors containing the five introns of *Reg1* were constructed, and designated as pGL3-intron 1, pGL3-intron 2, pGL3-intron 3, pGL3-intron 4 and pGL3-intron 5. All constructs were confirmed by Xho I/Hind III digestion and DNA sequencing.

### Luciferase assay

The SGC7901 gastric cancer cell line was plated on 96-well plates at a density of 2×10^3^ cells/well. The cells were transiently transfected with the above five luciferase reporter plasmids using Lipofectamine Plus system according to the manufacturer’s instructions (Invitrogen Life Technologies, Carlsbad, CA, USA). To evaluate the effect of gastrin (Sigma-Aldrich), the transfected cells were incubated with gastrin (1×10^−7^ mol/l) for 24 h after 48 h transfection. The luciferase activity of the transfected cells without or with gastrin incubation was measured by Luciferase Assay System (Promega Corporation, Madison, WI, USA) in a Glomax fluorescence detector (Promega Corporation) according to the manufacturer’s instructions. All assays were conducted in triplicate.

### Southwestern blotting

Using genomic DNA from human blood cells as a template, the five intron fragments of *Reg1* gene were cloned by PCR using the same primers (Table I) and identified by 1% agarose gel electrophoresis. Subsequently, the PCR products of the introns were randomly labeled with digoxin (Roche Diagnostics Corporation, Indianapolis, IN, USA) as a probe according to the manufacturer’s instructions. The sensitivity of the probes was detected with DNA dot blotting by using the DIG High Prime DNA Labeling and Detection Starter Kit I (Roche Diagnostics Corporation). To observe the effect of gastrin, the SGC7901 cells were cultured and incubated without or with gastrin (1×10^−7^ or 1×10^−8^ mol/l) for 24 h. Their nuclear proteins were subsequently extracted according to the manufacturer’s instructions (Pierce Biotechnology, Inc., Rockford, IL, USA). Protein concentration was determined by bicinchoninic acid (BCA) protein assay (BCA assay kit; Beyotime Institute of Biotechnology, Haimen, China) according to the manufacturer’s instructions. Nuclear proteins of the cells were subjected to sodium dodecyl sulfate-polyacrylamide gel electrophoresis (SDS-PAGE) in a 10% polyacrylamide gel. The proteins were blotted onto the PVDF membrane in a transfer buffer (Sigma-Aldrich). With the five intron fragments of *Reg1* as probes, the binding activity of each intron to nuclear proteins was detected as previously described (20). The density of each band was analyzed using Image-Pro Plus Version 6.0.

### Statistical analysis

All data are presented as the mean ± standard deviation (SD). Statistical analysis was performed using an unpaired two-tailed t test. P<0.05 was considered to indicate a statistically significant difference. Data analysis was performed using Statistical Product and Service Solutions software (version 15.0; SPSS, Inc., Chicago, IL, USA).

## Results

### Effects of introns of Reg1 on luciferase activity

**Figure 1:**
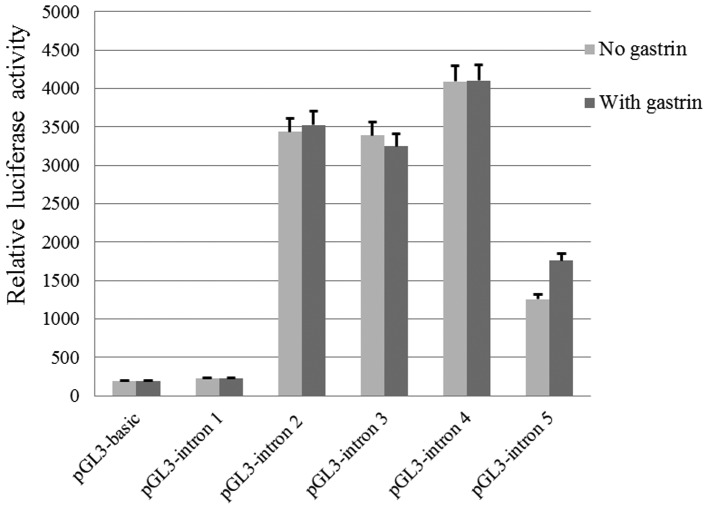
Comparative analysis of luciferase activity in transfected gastric cancer cells SGC7901. The cells transfected with pGL3-basic without any intron sequence were used as a control. The transfected cells were treated without or with 1×10^−7^ mol/l gastrin. The luciferase assay was performed as described in the methods section. The mean values and standard of three independent experiments are shown.

The five luciferase reporter vectors containing introns of the *Reg1* gene were identified by enzyme digestion and DNA sequencing. The results demonstrated that the sequences of inserted fragments were consistent with those of GenBank data with the correct direction of transcription. The relative luciferase activities in SGC7901 cells transfected with various recombinant plasmids are shown in Fig. 1.

The relative luciferase activities of cells transfected with pGL3-intron 2, pGL3-intron 3, pGL3-intron 4, and pGL3-intron 5 were significantly higher when compared with that of pGL3-Basic (P<0.05). No statistically significant difference in luciferase activity was identified between cells transfected with pGL3-intron 1 and those transfected with pGL3-Basic, which was used as the control (P>0.05). After gastrin incubation, no significant difference in luciferase activity was identified between cells transfected with any of the above recombinant plasmids (P>0.05).

### Transcription factors (Tfs) binding activity to five introns

**Figure 2:**
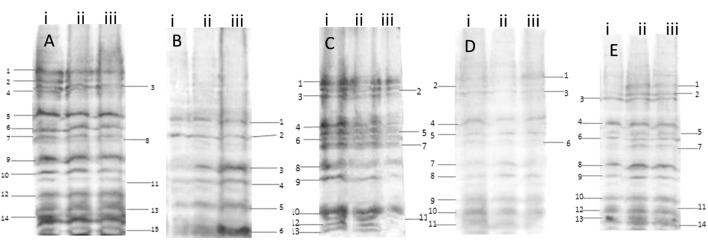
Analysis of transcription factors by Southwestern blotting with the five introns of the *Reg1* gene as probes: (A) intron 1, (B) intron 2, (C) intron 3, (D) intron 4 and (E) intron 5. Lane i, SGC7901 cells without gastrin incubation, as a control; lane ii, SGC7901 cells incubated with 1×10^−8^ mol/l gastrin; lane iii, SGC7901 cells incubated with 1×10^−7^ mol/l gastrin.

**Figure 3:**
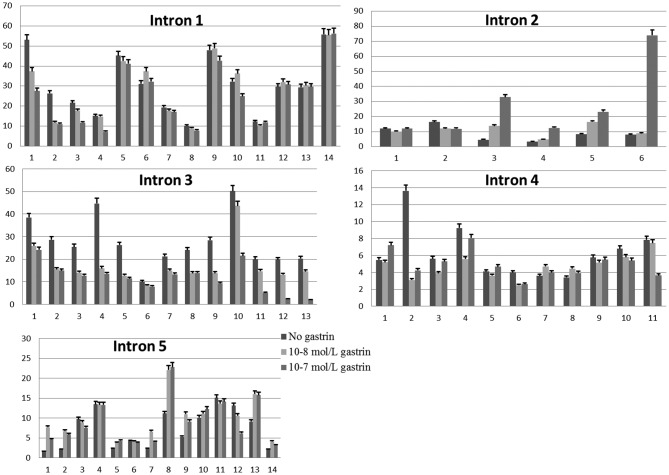
Quantitative analysis of effects of gastrin on transcription factors binding activity to five *Reg1* introns, performed by densitometric analysis of the bands. Data are presented as percentages of control values (mean ± standard deviation; n=3).

The five intron fragments were cloned by PCR and labeled with digoxin. The sensitivity of the five intron probes was 1 pg/μl, which was effective for detection. Results of the analysis of transcription factors through Southwestern blotting are shown in Figs. 2 and 3. For the probe of intron 1, 15 major bands of Tfs with different molecular weights were detected. Following gastrin treatment, the density of certain bands was evidently changed. Compared with the control group (without gastrin treatment), the density of bands 1, 2, 3, 4 and 7 was decreased significantly, and that of band 6 was increased significantly (P<0.05). For the cells incubated with 10^−8^ mol/l gastrin, the density of bands 1, 3, 4, 6, 9 and 10 was increased significantly when compared with that of the cells incubated with 10^−7^ mol/l gastrin (P<0.05).

With the intron 2 fragment as a probe, six different major bands of Tfs were detected. Following gastrin treatment, the density of band 2 was decreased significantly, and bands 3, 4, 5 and 6 were increased significantly (P<0.05). The density of bands 3, 4, 5 and 6 was increased significantly in the cells incubated with 10^−7^ mol/l gastrin compared with that of 10^−8^ mol/l gastrin (P<0.05).

The results of the intron 3 probe detection identified 13 different major bands of Tfs. Compared with the control group, the density of all 13 bands in the experimental groups was decreased significantly (P<0.05). The density of bands 4, 9, 10, 11, 12 and 13 were increased significantly in the cells incubated with 10^−8^ mol/l gastrin compared with that of the cells incubated with 10^−7^ mol/l gastrin (P<0.05).

The result of the intron 4 probe detection showed 10 different major bands of Tfs. Compared with the control group, the density of bands 2, 4, 6 and 11 were decreased significantly, and the density of bands 7 and 8 were increased significantly (P<0.05). When compared with those incubated with 10^−8^ mol/l gastrin, the density of bands 1, 3 and 4 was increased significantly for cells incubated with 10^−7^ mol/l gastrin, and that of band 11 was decreased significantly (P<0.05).

The result of the intron 5 probe detection showed 14 different major bands of Tfs. Compared with the control group, the density of band 12 was decreased significantly, and that of bands 1, 2, 5, 7, 8, 9 and 13 was increased significantly (P<0.05). The density of band 1, 7, 9 and 12 was increased significantly in the cells incubated with 10^−8^ mol/l gastrin compared to those incubated with 10^−7^ mol/l gastrin (P<0.05).

## Discussion

Human *Reg1* gene is a single copy gene that is located on chromosome 2 and is composed of 6 exons and 5 introns (21). One of the most well-documented effects of Reg1 is on the proliferation of acinar and islet cells of the pancreas. The expression of *Reg1* is increased in regenerating or hyperplastic islets. In addition to its islet proliferation- and regeneration-promoting effects, tumor-promoting activity of Reg1 protein has also been reported (22). Aberrant Reg expression has been detected in tissues from colorectal carcinoma and gastric cancer (23,24). In gastric cancer tissues, expression of *Reg1* gene is associated with patient survival and numbers of metastatic lymph nodes (25). Reg1-deficient mice have normal gastric development, however Reg1 promotes gastric mucosal growth and restoration with gastrin. Reg1 and gastrin may synergistically regulate gastric mucosal proliferation during certain pathological settings, such as wound healing (16). The proliferative efficiency of gastric cancer cell line SGC7901 decreases significantly following *Reg1* knock-down and incubation with gastrin (26). It has been suggested that *Reg1* may be a critical downstream gene in the process of gastrin stimulated gastric cancer development. To further understand the molecular mechanism by which gastrin stimulates the expression of *Reg1* in gastric cancer cells, the *cis-*regulatory function of the introns of *Reg1* and the relationship with gastrin were explored.

In recent years, studies have shown that introns of eukaryotic genes can promote transcriptional efficiency (27). The ability of introns to stimulate gene expression is an extensively investigated subject area for a wide range of organisms, including mammals and nematodes. Introns may act as transcriptional enhancers or alternative promoters, depending on *cis*-elements located within the intron spanning sequence (28). For example, uncoupling protein (*Ucp*) 2 and 3 expression is activated by the peroxisome proliferator-activated receptors (PPARs). The most prominent PPARγ binding site in the *Ucp2* and *Ucp3* loci was identified in intron 1 of the *Ucp3* gene and was the only site that facilitates PPARγ transactivation of a heterologous promoter. The transactivation of *Ucp2* and *3* is mediated through this novel enhancer in *Ucp3* intron 1 (29). A conserved Smad-binding element (SBE1) in intron 1 of the follistatin gene can also regulate the expression of this gene (30).

The present study demonstrated that the introns of the *Reg1* gene exhibited *cis*-regulating function, with the exception of intron 1, indicating that introns 2, 3, 4 and 5 of *Reg1* may contain *cis*-regulatory elements. It has been reported that a C-rich region of the rat *Reg1* promoter is critical for gastrin-stimulated *Reg* expression (15). It is hypothesized that *cis*-regulatory elements in introns and promoters of *Reg1* may synergistically regulate the gene expression. The present study also showed that, following gastrin incubation, the luciferase activity was not significantly different, which appeared to indicate that gastrin had no effect on regulating *Reg1* gene expression. However, in physiological conditions, gene expression is regulated by interaction of promoter and intron. The luciferase activity was detected in the cells transfected with only a single intron, which may contribute to the negative results obtained in the current study.

In eukaryotic cells, transcriptional regulation is executed by the interaction between *trans-*acting factors and *cis*-acting regulatory elements. *Trans-*acting factors are also known as transcription factors, and can recognize and bind to specific *cis*-acting elements. For example, the *cis*-elements of human ubiquitin C, able to bind *in vitro* the ubiquitous Sp1 and YY1 transcription factors, are involved in the stimulation of reporter gene transcription (31). In the present study, the effect of gastrin on Tfs that bind to the *cis*-acting regulatory elements in introns of the *Reg1* gene in gastric cancer cells was also explored by Southwestern blotting. The results revealed multiple Tfs binding to the five introns of *Reg1*, which suggested that the introns may function via binding to their Tfs.

The direct effect of intron-mediated transcriptional regulation is often referred to as ‘intron-mediated enhancement’ (IME) (28). IME requires the presence of an intron close to the 5′ end of the gene. It has been hypothesized that a promoter proximal to the 5′ splice site facilitates the recruitment of transcriptional machinery to the promoter, which includes transcription factors such as c-Jun and activating transcription factor 2 binding to the cyclic adenosine monophosphate response element site and, therefore, aids in the initiation of transcription (32–34). The precise mechanism of intron-dependent enhancement of transcription, however, remains unclear. It was recently reported that the inclusion of an intron in *INO1*, which is a nonintronic gene, resulted in the constitutive activation of the gene. In the presence of the intron, the promoter of *INO1* interacted with its terminator region to form a gene loop in yeast (35). The intronic *cis*-acting elements of the cystic fibrosis transmembrane conductance regulator gene (*CFTR*) interact with the *CFTR* promoter and contribute to the regulation of *CFTR* gene expression (36). In *Reg1*, the C-rich region in the gene promoter was found to be critical for the response to gastrin (15,37), and the expression of *Reg1* was controlled through separate promoter elements by gastrin (4).

The present study found that gastrin may alter the density of certain Tf bands, and that the density of some Tf bands was altered at different gastrin concentrations. This indicates that gastrin can alter the ability of Tfs to bind to the recognition sequences in introns to affect the formation of the transcriptional complex, and may be significant in the interaction between the promoter and introns to regulate the expression of *Reg1*. Although the first intron of *Reg1* has no *cis*-regulatory function, it can bind to at least 14 types of Tf. It was reported that the control of human *Ucp3* transcription in skeletal muscle is not solely conferred by the promoter, but depends on several *cis-*acting elements in intron 1, suggesting a complex association between the promoter and intronic sequences (38). Intron removal, or replacement with a heterologous chimeric intron, caused a significant reduction in promoter activity (27). The results of the current study suggest that intron 1 of the *Reg1* gene may function only as a mediator in the formation of multiple molecule complexes for regulating gene expression.

In conclusion, our data demonstrate that introns of *Reg1* can bind to many transcription factors and enhance gene expression. However, we still did not identify the precise transcription factors. The hormone gastrin can influence the ability of Tf binding to introns. Gastrin may regulate *Reg1* gene expression by binding to the transcription factors to form a multiple molecule complex. Future research on the interaction of the promoter and introns of the *Reg1* gene and identifying the transcription factors that bind to the introns of *Reg1* gene will be useful in elucidating the mechanism of *Reg1* gene expression.

## Figures and Tables

**Table I:** Polymerase chain reaction primer sequences for the five introns of the *Reg1* gene.

Introns	Forward primer 5′-3′	Reverse primer 5′-3′	PCR products length, bp	Tm, °C
Intron1	CCAACTCAGACTCAGCCAAC	CATGCTGAGCTGCAATGAAT	376	50
Intron2	GCTGATCTCCTGCCTGATGT	AACTCTGTCTGGGCCTCTTG	700	55
Intron3	TGCCTATCGCTCCTACTGCT	AGGTTGCCCGAATTCATGT	400	55
Intron4	CCTTTGTGGCCTCACTGATT	CAATGCCCCAGGACTTGTAG	850	52
Intron5	CTGTGTGAGCCTGACCTCAA	CAAGGCACATCCTTCCATTT	245	55

Tm, annealling temperature.
